# Brugada Syndrome and Kawasaki Disease in a Two-Year-Old: A Case of Double Jeopardy

**DOI:** 10.7759/cureus.75001

**Published:** 2024-12-02

**Authors:** Jeffery O Boateng, Ilana Stein, Nicholas Hidalgo

**Affiliations:** 1 Pediatrics, St. Barnabas Hospital Health System, Bronx, USA; 2 Pediatric Cardiology, Children's Hospital at Montefiore, Bronx, USA

**Keywords:** brugada syndrome, channelopathy, fever, kawasaki disease, ventricular arrhythmia

## Abstract

Brugada syndrome (BrS) is a genetic channelopathy that may predispose to ventricular arrhythmia. It is inherited as an autosomal dominant pattern with incomplete penetrance. Fever can unmask Brugada syndrome in children who have a genetic predisposition. Kawasaki disease is not known to be associated with Brugada syndrome; however, it may lead to cardiac complications such as coronary artery abnormalities and myocardial inflammation. We report a case of a two-year-old who was diagnosed with Kawasaki disease and subsequently developed Brugada syndrome.

## Introduction

Brugada syndrome (BrS) is linked with variant mutations in the *SCN5A* gene located on the short arm of chromosome 3, resulting in a reduction of sodium influx through the voltage-gated sodium channels of the cardiac myocyte. Other multiple genes found on chromosome 6 have also been identified in genotype-positive patients [1]. Clinically, BrS is diagnosed based on three electrocardiogram (ECG) patterns. The diagnostic type 1 pattern is characterized by coved ST segment elevation > 0.2 mV, followed by a negative T wave, with little or no isoelectric separation in the right precordial leads of a standard 12-lead ECG [2]. The risk of symptomatic disease including ventricular arrhythmia and sudden cardiac death increases with age and is exacerbated by various factors, including fever, or certain drugs [3]. For the last 30 years, our knowledge of BrS has grown significantly; however, very little is known about this phenomenon in the pediatric population [4]. BrS ECG pattern co-existing with Kawasaki disease, a vasculitis that is commonly accompanied by myocarditis, may present a peculiar risk for pediatric patients [5]. This case describes a patient with BrS ECG pattern, unmasked by high fever in the setting of Kawasaki disease.

## Case presentation

A two-year-old female presented to the clinic with six days of persistent fever, maximum temperature of 39.4°C, rash, poor feeding, sleeping more than usual, and no urine production for more than 12 hours. Prior to this visit, she was seen in the emergency unit on the third day of illness with a generalized rash involving the extremities for which she was managed symptomatically. Her family history was significant for a history of arrhythmia in her father, and her paternal uncle had an implantable cardioverter defibrillator (ICD) placed when he was 30 years old and passed at 50 years from unclear etiology.

In the clinic, she appeared tired and poorly perfused, and her vitals showed a heart rate of 149 bpm, temperature of 39.9°C, respiratory rate of 26 per minute, oxygen saturation of 99%, and blood pressure of 91/46 mmHg. On examination, she was found to have conjunctival injection, perioral skin peeling with dry cracked lips, pharyngeal erythema and strawberry tongue, swollen hands and feet, and faintly disseminated macular rash worse around the diaper area. A bolus of isotonic intravenous fluids was administered, and she was admitted to the pediatric intensive care unit (ICU).

**Figure 1:**
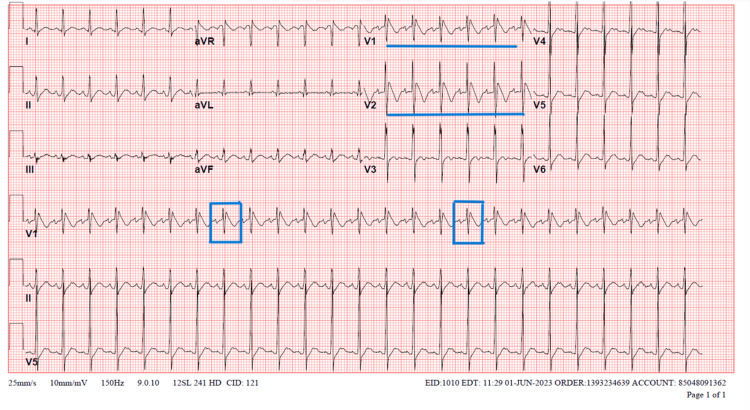
ECG of the patient showing coved ST segment elevation in leads V1 and V2 significant for Brugada ECG pattern ECG: electrocardiogram

On admission, initial laboratory results showed elevated inflammatory markers, anemia, hyponatremia with acute kidney injury, and sterile pyuria (Table 1). A baseline ECG done at the peak of fever showed “coved” ST segment elevation in lead V1 and V2 with T wave inversion, classic for BrS ECG pattern type 1 (Figure 1). The patient was started on high-dose aspirin daily and received the first intravenous immunoglobulin (IVIG) infusion after initial resuscitation. The patient was kept on a cardiac monitor to detect any arrhythmia in the setting of a persistent BrS ECG pattern. A baseline echocardiogram showed left main and proximal left anterior descending coronary artery ectasia, trivial-to-mild mitral regurgitation with preserved function, and no pericardial effusion.

**Table 1:** Initial laboratory results of the patient at presentation CRP: C-reactive protein, ESR: erythrocyte sedimentation rate, WBC: white blood cell count, MCV: mean corpuscular volume, MCH: mean corpuscular hemoglobin, MCHC: mean corpuscular hemoglobin concentration, BUN: blood urea nitrogen, Cr: creatinine

Laboratory parameter	Result	Normal range
Inflammatory markers		
CRP	30.3 mg/dL	<0.8 mg/dL
ESR	95 mm/hour	0-10 mm/hour
Ferritin	500.4 ng/mL	10.0-150.0 ng/mL
D-dimer	5.6 ug/mL	0.27-0.50 ug/mL
Pro-calcitonin	31.4 ng/mL	<0.10 ng/mL
Complete blood count		
WBC	27.4 k/uL	6.0-17.5 k/uL
Hemoglobin	9.4 g/dL	11.7-13.8 g/dL
Hematocrit	29.5%	34.0%-40.0%
MCV	81.0 fL	75.0-87.0 fL
MCH	25.8 pg	24.0-30.0 pg
MCHC	31.9 g/dL	31.0-37.0 g/dL
Kidney function test		
Sodium	124 mEq/L	135-145 mEq/L
Chloride	93 mEq/L	98-108 mEq/L
Potassium	4.0 mEq/L	3.5-4.5mEq/L
BUN	50 mg/dL	5-20 mg/dL
Cr	0.84 mg/dL	<0.50 mg/dL
Urinalysis		
Color	Dark yellow	Yellow
Appearance	Cloudy	Clear
Specific gravity	1.015	1.006-1.029
pH	6.0	5.0-8.0
Protein	100 mg/dL	Negative
Blood	Trace	Negative
Nitrite	Negative	Negative
Leucocyte esterase	Trace	Negative

During her ICU stay, medications known to trigger BrS such as metoclopramide, diphenhydramine, ketamine, propofol, and antiarrhythmic drugs were avoided. Acetaminophen was given as needed to control fever. Thirty-six hours after the first IVIG infusion, the patient continued to spike fevers, which warranted a second IVIG infusion for refractory Kawasaki disease. Although she continued to exhibit the BrS pattern on ECG, even when afebrile, no ventricular tachycardia or syncope was witnessed. The patient became afebrile after the second dose of IVIG, and the BrS ECG resolved.

By day 4 of her ICU stay, clinical status had improved, all inflammatory markers were downtrending, and care was downgraded to the pediatric floor. Repeat echocardiogram four days post resolution of symptoms showed normal coronary artery dimensions. The patient was then discharged on low-dose aspirin, and the family was provided with detailed education on drugs to avoid in patients with BrS and aggressive management of fever in the future.

During her outpatient follow-up a week after discharge, both ECG and echocardiogram were normal, and the patient was well-appearing. Full gene sequencing using next-generation sequencing (NGS) technology was performed, which showed a variant of uncertain significance mutation in the *AKAP9* gene. This genetic testing has >99% analytical sensitivity and specificity for single nucleotide variants, insertions, and deletions < 15 bp in length and duplications. Although she has been seen in the emergency room for viral illnesses since this admission, there has not been any recording of the BrS pattern on her ECG to our knowledge.

## Discussion

BrS ECG pattern may be spontaneously seen but is commonly unmasked by antiarrhythmic, psychotropic, or anesthetic medications, ischemia, or, like in our case, fever [6,7]. Prior studies have suggested that there is increased sensitivity of the sodium channels as core temperature rises, which not only leads to ECG changes but also increases the risk of ventricular arrhythmias [8]. In children, the most important precipitating event to symptomatic disease is fever [9]. Similarly, the patient’s BrS ECG pattern was noticed when she had the highest measured fever, which is supported by the phenomenon of the effect of fever on the sodium channels. Although other case reports have reported BrS unmasked by fever, to the best of our knowledge, this is the first case of BrS co-existing with another disease with a cardiac sequela, Kawasaki disease.

Kawasaki disease is the leading cause of acquired heart disease among the pediatric population in developed countries. It is diagnosed clinically by the presence of fever lasting more than five days and four out of the following five symptoms: conjunctival injection, oral mucous membrane changes, peripheral extremity changes, polymorphous rash, and cervical lymphadenopathy, all of which were seen in our patient. Additionally, Kawasaki disease is usually accompanied by laboratory abnormalities observed in the patient such as elevated inflammatory markers, sterile pyuria, anemia, transaminitis, and electrolyte derangement [10]. The inflammatory process associated with Kawasaki disease may involve the coronary arteries, the myocardium, or, less commonly, the cardiac valves. Sequela from this process may include coronary artery aneurysm, which may lead to thrombosis and/or myocardial infarction, and myocarditis, which may lead to myocardial scarring [11]. These sequelae may serve as a nidus for future arrhythmias, even in the normal heart. Although the patient developed a cardiac complication putting her at risk for arrhythmia, she maintained a sinus rhythm throughout her ICU stay. It is important to recognize that in patients who have a baseline increased risk of arrhythmias such as BrS, having another acquired predisposition to arrhythmias offers an added risk of eventually having polymorphic ventricular tachycardia, ventricular fibrillation, or even sudden cardiac death in the immediate or long term. Fortunately, our patient did not experience this outcome.

Currently, there are no BrS-specific guidelines in the pediatric population largely because of the rarity of symptomatic disease, paucity of data on prevalence, diagnostic criteria, and natural history of the disease [9]. However, as highlighted by this case, children with a genetic predisposition may have an increased risk due to the commonality of febrile illnesses and the risk of arrhythmogenic conditions such as Kawasaki disease. Certainly, these patients will require a more robust follow-up and discharge planning compared to the general population.

## Conclusions

In genetically predisposed patients whose BrS is unmasked by fever, having another cardiac affection increases the theoretical risk for ventricular tachyarrhythmia. Pediatric emergency physicians should therefore have a low threshold for ECG in a febrile child with a family history of arrhythmia. Furthermore, pediatricians and pediatric cardiologists alike should be mindful of the increased theoretical risk of arrhythmia in patients with BrS and associated possible cardiac sequelae of inflammatory conditions, as this may influence monitoring and long-term care and follow-up.
